# Comparison of dexmedetomidine-propofol and ketamine-propofol administration during sedation-guided upper gastrointestinal system endoscopy

**DOI:** 10.1097/MD.0000000000023317

**Published:** 2020-12-04

**Authors:** Arzu Esen Tekeli, Ali Kendal Oğuz, Yunus Emre Tunçdemir, Necat Almali

**Affiliations:** a Department of Anesthesiology and Reanimation, Van Yuzuncu Yil University School of Medicine; b Department of General Surgery, Van Yuzuncu Yil University School of Medicine, Van, Turkey.

**Keywords:** dexmedetomidine, ketamine, propofol, sedation, bispektral indeks

## Abstract

**Background:**

Dexmedetomidine and ketamine popular sedative agents that result in minimal respiratory depression and the presence of analgesic activity. We aimed to compare the effectiveness and safety of a dexmedetomidine-propofol combination and a ketamine-propofol combination during upper gastrointestinal system endoscopy.

**Methods:**

The study commenced after receiving approval from the local ethics committee. Patients between 18 and 60 years in the American Society of Anesthesiologists (ASA) I and II groups were included. Patients who had severe organ disease, who had allergies to the study drugs, and who refused to participate were excluded. Cases were randomized into a dexmedetomidine-propofol group (Group D, n = 30) and a ketamine-propofol group (Group K, n = 30). Cardiac monitoring, peripheral oxygen saturation, and bispectral index (BIS) monitoring were performed. Group D received 1 mg/kg dexmedetomidine + 0.5 mg/kg propofol intravenous (IV) bolus, 0.5 μg/kg/h dexmedetomidine + 0.5 mg/kg/h propfol infusion. Group K received 1 mg/kg ketamine + 0.125 mL/kg propofol iv bolus, 0.25 mg/kg/h ketamine + 0.125 mL/kg/h propfol infusion. Patients were followed up with a Ramsay Sedation Scale (RSS) of ≥4. Means, standard deviations, lowest and highest frequency values, and ratio values were used for descriptive statistics, and the SPSS 22.0 program was used for statistical analyses.

**Results:**

In Group K, recovery time and mean blood pressure (MBP) values were significantly shorter. Furthermore, coughing rate, pulse, and BIS values were higher than in Group D (*P* < .05). Although there were no significant differences between the groups in terms of endoscopic tolerance and endoscopist satisfaction, we observed that the dexmedetomidine group experienced more comfortable levels of sedation.

**Conclusion:**

Dexmedetomidine-propofol and ketamine-propofol combinations may be suitable and safe for endoscopy sedation due to their different properties. It was observed that the dexmedetomidine-propfol combination was superior in terms of sedation depth and that the ketamine-propofol combination was superior in terms of early recovery. As a result, we suggest the dexmedetomidine-propofol combination for upper gastrointestinal system endoscopy sedation due to hemodynamic stability and minimal adverse effects.

## 1 Introduction

It has been observed that endoscopic procedures are gradually increasing all over the world in screening, diagnosis, and treatment of diseases.^[1]^ However, anxiety, pain, fear, and digestive tract reactions may cause compliance problems, adverse cardiovascular and respiratory events, and unwanted injuries during endoscopy.^[2]^ Conscious sedation is important in endoscopy for patient and physician comfort.

Several different sedatives and analgesics can be used in gastrointestinal endoscopic procedures to achieve appropriate sedation levels. Dexmedetomidine is a highly selective α2 adrenergic agonist. Besides its sedative effect, it also displays analgesic efficacy. Whereas minimal respiratory depression is an important advantage, it can cause bradycardia and hypotension. It is synergistic with other sedatives and opioids, and it should be used with caution. Dexmedetomidine is used for mild to moderate sedation. Adding propofol for deeper sedation may be preferred.^[3]^ Propofol, a phenolic derivative, has sedative and hypnotic effects mediated by the gamma amino butyric acid (GABA) receptor. It has no analgesic effect, and it is highly lipophilic and therefore quickly crosses the blood–brain barrier, providing early onset of action and rapid recovery. The main disadvantage of propofol is respiratory and cardiovascular depression because of the risk of rapidly induced deep sedation.^[4]^ Ketamine, an N-methyl-D-aspartate (NMDA) receptor antagonist, is a dissociative anesthetic with analgesic properties. It maintains airway muscle tone.^[5]^ With the combined use of ketamine and propofol (ketofol), the required amount of medication is reduced, and a stable structure is formed. It provides rapid effect, but recovery can be delayed.^[6]^ The targeted sedation level varies depending on the patient and the procedure. For a safe, comfortable, and technically successful endoscopic procedure, sedative doses must be titrated. During sedation, monitoring of pulse, blood pressure, respiratory status, oxygen saturation, cardiac electrical activity, and sedation level are recommended.^[7]^ In the clinical monitoring of sedation levels, the Ramsay sedation scale (RSS) and bispectral Index (BIS) (which ranges between 0 = deep and coma − 100 = completely awake) are widely used today.^[8,9]^ It has been reported that loss of consciousness occurs at BIS values between 60 and 80.^[10]^

In this study, we compared dexmedetomidine-propofol and ketamine-propofol combinations used for sedation in the upper gastrointestinal system endoscopy in terms of efficacy and safety. We aimed to show that the dexmedetomidine-propofol combination could have similar or superior safety and efficacy with ketamine propofol combination, in terms of patient comfort, endoscopist satisfaction, side effects, recovery times and effects on hemodynamic and respiratory functions.

## 2 Methods

### 2.1 Study design and participants

*Inclusion criteria:* Patients classified into the American Society of Anesthesiologist (ASA) I and II group. These included 60 patients between the ages of 18 and 60 who were scheduled to have upper gastrointestinal system (UGIS) endoscopy with sedation were included in the study. Patients were divided into two groups (each one includes 30 patients) with closed envelope draw.

*Exclusion criteria:* Those who had severe heart, lung, and liver disease, kidney failure, bleeding diathesis, fever, infection, electrolyte disorders, such as hypokalemia and hypocalcaemia, acid–base disorder, hypothermia, allergy to drugs to be used, and those who refused to participate were excluded.

### 2.2 Ethics statement

This prospective randomized, controlled, and single-center clinical interventional study was approved by the local ethics committee of Van yuzuncu Yil University. The study protocol was approved on October 18, 2019 date, and the decision number was 02. The protocol of this study was based on the ethical standards of the Declaration of Helsinki. All participants were informed about the study and their written consents were obtained.

### 2.3 Sedation protocol

Sedation was administered to participants with dexmedetomidine-propofol (Group D, n = 30), ketamine-propofol (Group K, n = 30) according to the group they were assigned to. Cardiac monitoring (electrocardiogram = ECG), non-invasive arterial pressure monitoring, peripheral oxygen saturation (SpO2) monitoring, and BIS monitoring were performed. The RSS, patient satisfaction, endoscopist satisfaction, patient tolerance, and side effects were recorded. Group D received 1 μg/kg dexmedetomidine + 0.125 mL/kg propofol intravenous (IV) bolus, 0.5 μg/kg/h dexmedetomidine + 0.125 mL/kg/h propfol infusion. Group K received 1 mg/kg ketamine + 0.125 mL/kg propofol iv bolus, 0.25 mg/kg/h ketamine + 0.125 mL/kg/h propfol infusion.

### 2.4 Assessment

Sedation level was evaluated at 1 to 3 min intervals. All procedures took 5 to 6 min, and the infusion rate followed was RSS ≥ 4 (Table 1). In the case of RSS < 4, propofol addition was planned, but this was not needed. Systolic blood pressure, diastolic blood pressure, mean blood pressure, heart rate, periepheric oxygen saturation, and sedation depth were monitored.

**Table 1:** Ramsay Sedation Scale.

Point	Clinical evaluation
1	Anxious and agitated or restless, or both
2	Cooperative, oriented, and tranquil
3	Responds to commands only
4	Brisk response to light glabellar tap or loud auditory stimulus
5	A sluggish response to light glabellar tap or loud auditory stimulus
6	No response

### 2.5 Statistical analysis

*Sample size calculation:* According to the previous study, it was observed that standard deviation for BIS value ranged from 1 to 15.^[11]^ Thus, in this study, standard deviation was taken as 8 for BIS value. In addition, for the 0.05 type I error rate, *Z* value and effect size were assumed 1.96 and 3, respectively. Based on this information and according to the equation of sample size calculation (n = *Z*^2^ σ^2^/*d*^2^), the minimum sample size was found 27.

Descriptive statistics for the continuous variables were presented as Mean, Median, and Standard deviation, while count and percentages for categorical variables. Normality of continuous variables was tested by Kolmogorov–Smirnov test. According to whether providing of normality assumption, Mann–Whitney *U* test was used to compare independent groups. In addition, Wilcoxon test was also used to compare dependent groups. Chi-square test was performed to determine the relationship between categorical variables. Statistical significance level was considered as 5% and SPSS (ver: 22) statistical program was used for all statistical computations.

## 3 Results

### 3.1 Patient characteristics

**Figure 1:**
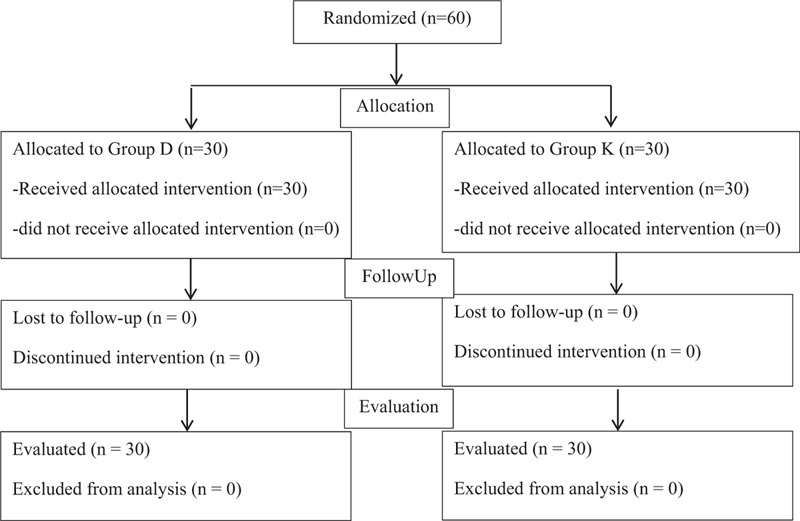
Trial profile.

Sixty patients, ASA I and II between the ages of 18 and 60, who were scheduled for upper gastrointestinal system endoscopy with sedation, were included in the study (Fig. 1).

### 3.2 Study outcomes

There was no significant difference between Group D and Group K in terms of age, gender, height, weight, body mass index (BMI), and ASA classification (*P* > .05), as portrayed in Table 2.

**Table 2:** Demographic characteristics.

		Group D		Group K			
		Mean ± S.d./n-%		Mean ± S.d./n-%		*P*	
Age		36.6 ± 13.1		36.2 ± 10.5		.885	^m^
Gender	*Female*	38	63.3%	39	65.0%	.849	X^2^
	*Male*	22	36.7%	21	35.0%		
Height		163.6 ± 8.0		165.8 ± 9.2		.123	^m^
Weight		67.1 ± 13.0		70.2 ± 15.6		.418	^m^
BMI		25.1 ± 4.7		25.5 ± 5.1		.686	^m^
ASA	*I*	36	60.0%	36	60.0%	.910	X^2^
	*II*	23	38.3%	24	40.0%		

^m^ Mann–Whitney *u* test/Chi-square test. ASA = American Society of Anesthesiologists; BMI =  body mass index.

There was no significant difference between Group D and Group K in terms of RSS values, patient tolerance, patient satisfaction, and endoscopist satisfaction (*P* > .05) (Table 3). However, even though there was no statistically significant difference, verbal satisfaction in Group D was excessive.

**Table 3:** Evaluations during endoscopy.

		Group D		Group K			
		Mean ± S.d./n-%		Mean ± S.d./n-%		*P*	
RSS		5.2 ± 0.8		4.9 ± 0.7		.067	^m^
Patient's discomfort score	*No*	36	60.0%	28	46.7%	0200	X^2^
	*Mild reaction*	20	33.3%	28	46.7%		
	*Verbal defense*	0	0.0%	2	3.3%		
	*Manual defense*	4	6.7%	2	3.3%		
Patient's Tolerance to endoscopy	*No*	35	58.3%	30	50.0%	.463	X^2^
	*Mild reaction*	21	35.0%	26	43.3%		
	*Verbal defense*	0	0.0%	1	1.7%		
	*Manual defense*	4	6.7%	3	5.0%		
Endoscopist satisfaction	*Excellent*	21	35.0%	14	23.3%	.334	X^2^
	*Good*	28	46.7%	35	58.3%		
	*Not bad*	10	16.7%	8	13.3%		
	*Bad*	1	1.7%	3	5.0%		

^m^ Mann–Whitney *U* test/Chi-square test.

Indications for application of UGIS endoscopy did not differ between the groups (*P* > .05). Nausea, vomiting, and allergic reaction rates were similar for both groups (*P* > .05). In Group K, mild coughing was detected in 30% of patients, and severe coughing in 5%, and this was found to be statistically significant (*P* < .05). It was also noted that the recovery time in Group K was significantly shorter than in Group D (*P* < .05). In Group D, the rate of patients whose recovery times were below 15 min was 11.7%, whereas it was 35% in Group K (Table 4).

**Table 4:** Indications, complications and recovery times.

		Group D		Group K			
		Mean ± S.d./n-%		Mean ± S.d./n-%		*P*	
Indication	Dysphagia	16	26.7%	9	15.0%	.211	X^2^
	Reflux	6	10.0%	11	18.3%	.266	X^2^
	Dyspepsia	32	53.3%	36	60.0%	.471	X^2^
	Weight loss	11	18.3%	7	11.7%	.489	X^2^
Complication							
Apnea		0	0.0%	3	5.0%	.244	X^2^
Desaturation		6	10.0%	12	20.0%	.125	X^2^
Nausea		0	0.0%	0	0.0%	1.000	X^2^
Vomiting		0	0.0%	0	0.0%	1.000	X^2^
Allergy		0	0.0%	0	0.0%	1.000	X^2^
Coughing	*No*	53	88.3%	39	65.0%	***.005***	X^2^
	*Mild*	7	11.7%	18	30.0%		
	*Severe*	0	0.0%	3	5.0%		
Recovery time	<15 min	7	11.7%	21	35.0%	***.002***	X^2^
	15–30 min	35	58.3%	35	58.3%		
	30–60 min	16	26.7%	4	6.7%		
	>60 min	2	3.3%	0	0.0%		

^m^ Mann–Whitney *U* test/Chi-square test. Bold italic values signifies statistically significance.

During the presedation period (at 0, 3, and 5 min) and during the sedation period, systolic, diastolic, and mean blood pressure values were monitored. Values of presedation time, third minute and fifth minute systolic blood pressure, and mean blood pressure did not differ between the groups (*P* > .05). In intra-group evaluations, it was observed that systolic and mean arterial pressure values in Group K were significantly lower than the presedation period (*P* < .05). Diastolic blood pressure values did not differ between groups (*P* > .05), as shown in Table 5.

**Table 5:** Blood pressure changes within and between groups.

	Group D	Group K	
	Mean ± S.d./n-%	Mean ± S.d./n-%	*P*
*Systolic blood pressure*			
Presedation	135.3 ± 18.0	133.3 ± 15.4	.651^m^
0 min	134.3 ± 20.6	121.2 ± 14.7	*.**000***^**m**^
Intra-group change p	0.574^w^	*0.**000***^**w**^	
3 min	124.1 ± 18.5	123.6 ± 13.6	.951^m^
Intra-group change p	*0.**000***^**w**^	*0.**000***^**w**^	
5 min	127.0 ± 19.2	125.6 ± 15.8	.956^m^
Intra-group change p	*0.**032***^**w**^	*0.**015***^**w**^	
*Diastolic blood pressure*			
Presedation	80.8 ± 14.6	79.2 ± 11.0	.562^m^
0 min	80.2 ± 14.8	77.2 ± 11.1	.160^m^
Intra-group change p	0.742^w^	0.142^w^	
3 min	77.2 ± 15.7	78.4 ± 11.3	.376^m^
Intra-group change p	0.056^w^	0.466^w^	
5 min	78.4 ± 13.7	81.9 ± 13.3	.381^m^
Intra-group change p	0.114^w^	0.589^w^	
*Mean blood pressure*			
Presedation	98.3 ± 14.6	97.1 ± 11.1	.747^m^
0 min	95.8 ± 15.1	90.6 ± 12.1	*.005*^m^
Intra-group change p	0.448^w^	*0.**000***^**w**^	
3 min	92.2 ± 16.6	92.7 ± 10.9	.406^m^
Intra-group change p	*0.**021***^**w**^	*0.**012***^**w**^	
5 min	92.6 ± 16.1	94.9 ± 13.6	.568^m^
Intra-group change p	0.117^w^	0.218^w^	

^m^ Mann–Whitney *U* test/ ^w^ Wilcoxon test. Bold italic values signifies statistically significance.

Heart rate, saturation (SpO2), and BIS value changes were also examined in both groups and between groups, and the results are demonstrated in Table 6.

**Table 6:** Intra and intergroup study characteristics.

	Group D	Group K	
	Mean ± S.d./n-%	Mean ± S.d./n-%	*P*
*Heart rate*			
Presedation	88.4 ± 17.7	86.5 ± 16.1	.592^m^
0 min	68.8 ± 16.3	86.1 ± 15.8	***.000***^m^
Intra-group change **p**	***0.000***^w^	0.603^w^	
3min	75.1 ± 17.8	88.6 ± 16.5	***.000***^m^
Intra-group change **p**	***0.000***^w^	0.137^w^	
5 min	74.7 ± 16.8	86.5 ± 15.1	***.002***^m^
Intra-group change **p**	***0.000***^w^	0.689^w^	
*SPO*_2_			
Presedation	97.8 ± 1.7	98.0 ± 2.2	.242^m^
0 min	96.5 ± 5.1	94.8 ± 10.1	.410^m^
Intra-group change **p**	***0.048***^w^	***0.000***^w^	
3 min	96.1 ± 4.7	95.4 ± 8.1	.756^m^
Intra-group change **p**	***0.030***^w^	***0.001***^w^	
5 min	97.6 ± 2.3	98.1 ± 1.6	.287^m^
Intra-group change **p**	0.714^w^	0.950^w^	
*BIS*			
Presedation	93.9 ± 11.3	95.8 ± 4.2	.404^m^
0 min	48.5 ± 16.0	56.2 ± 15.8	***.009***^m^
Intra-group change **p**	***0.000***^w^	***0.000***^w^	
3 min	45.7 ± 15.1	60.9 ± 16.1	***.000***^m^
Intra-group change **p**	***0.000***^w^	***0.000***^w^	
5 min	54.3 ± 15.5	63.7 ± 15.4	***.031***^m^
Intra-group change **p**	***0.000***^w^	***0.000***^w^	

BIS = Bispektral index, SPO_2_ =_ _peripheral oxygen saturation. ^m^ Mann–Whitney *U* test/ ^w^ Wilcoxon test. Bold italic values signifies statistically significance. The difference between the groups indicated in bold fond on the same line is statistically significant (*P* ≤ .05).

**Figure 2:**
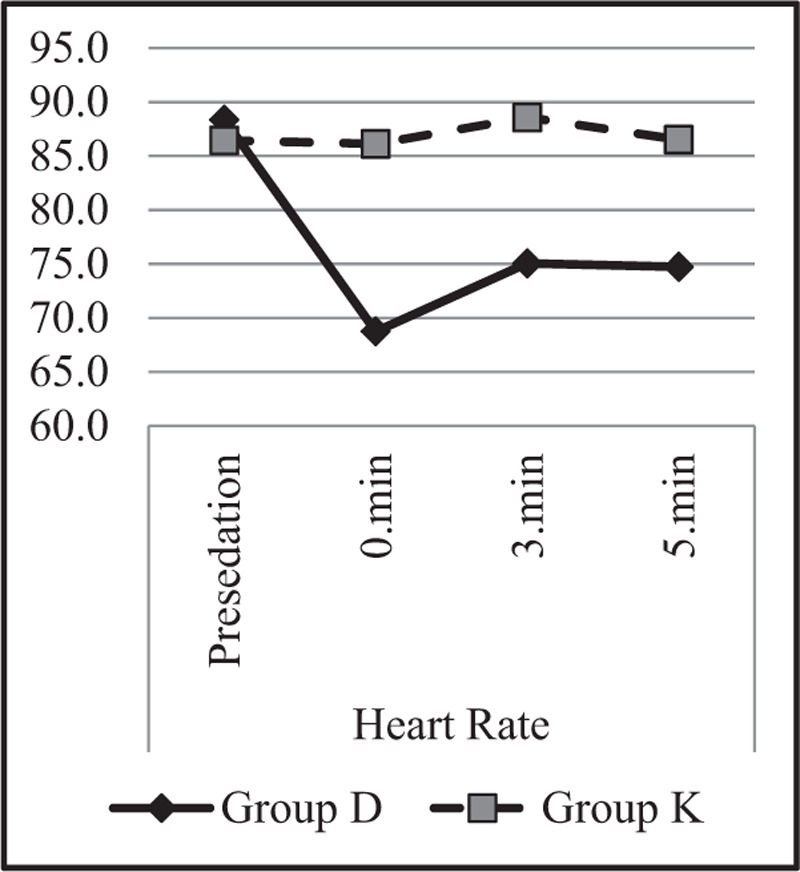
Heart rate changes.

The heart rate values of the presedation period did not differ between the groups (*P* > .05). Results revealed that that the 0, 3, and 5 min heart rate values were higher in Group K (*P* < .05). In the evaluation conducted in Group D, it was observed that the pulse values of 0, 3, and 5 min were significantly lower compared to the presedation period (*P* < .05), and this change was not observed in Group K (*P* > .05) (Fig. 2).

**Figure 3:**
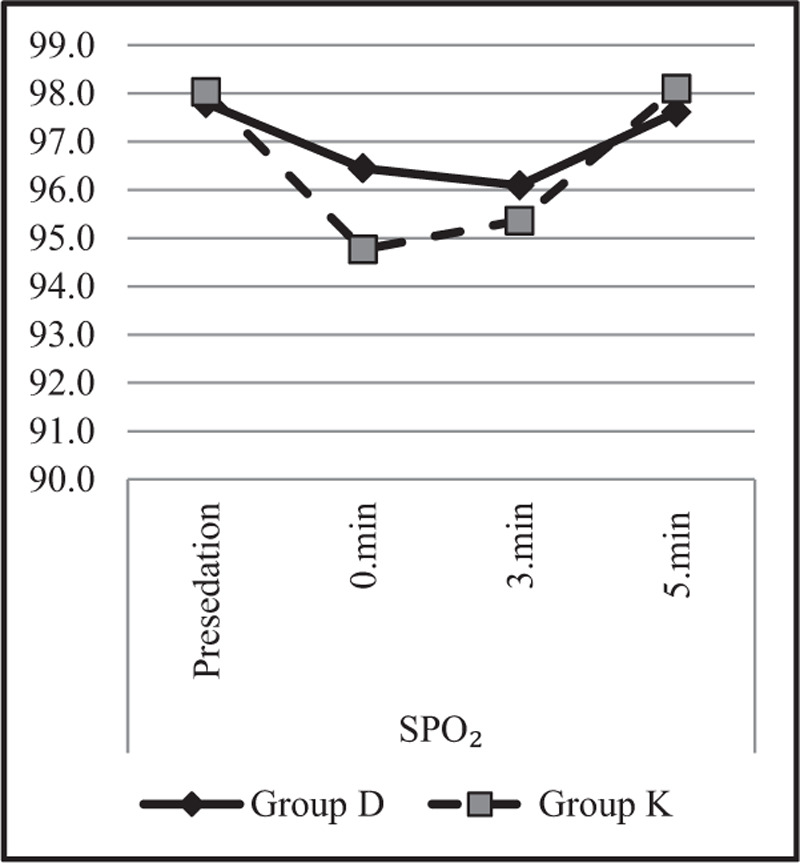
SpO_2_ changes.

In our study, no difference was observed in general adverse events between the two groups of drugs. A decrease was observed in heart rate, which did not adversely affect patients in Group D, and a decrease in saturation was observed in Group K. The lowest saturation value was recorded as 88% (two patients). Furthermore, 4 L of nasal oxygen supplement was administered to patients whose saturation was under 90%. It was observed that the values of presedation, 0, 3, and 5 min of SpO2 did not differ between the groups (*P* > .05). In addition, the SpO2 values at 0 and 3 min were lower in both groups compared to the presedation period (*P* < .05) (Fig. 3).

**Figure 4:**
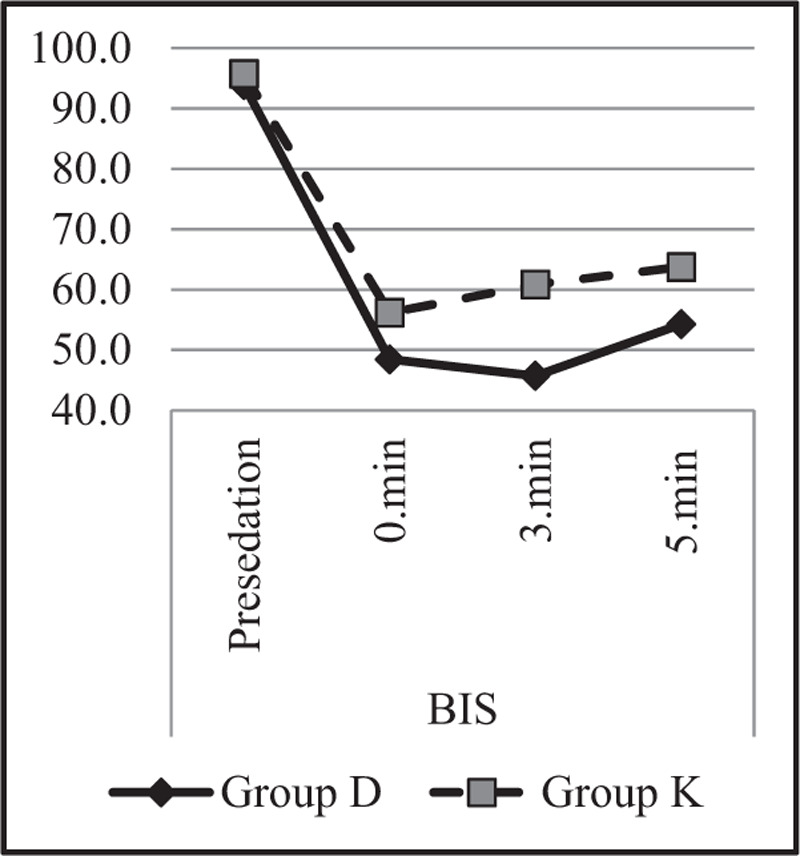
BIS changes.

It was noted that BIS values during the presedation period did not differ between the groups. In addition, the 0, 3, and 5 min BIS values in Group K were significantly higher than in Group D (*P* < .05). In both groups, BIS values of 0, 3, and 5 min were lower than the presedation period (*P* < .05) (Fig. 4).

## 4 Discussion

The importance of sedation has increased with the widespread use of endoscopic procedures. This study compared the effectiveness and reliability of two different moderate sedation regimens. Results suggested that the use of dexmedetomidine-propofol or ketamine-propofol during the upper gastrointestinal system endoscopy may be appropriate and safe. The combination of dexmedetomidine-propofol has been highlighted because of its better stable hemodynamics, better sedation level, preservation of saturation, and fewer side effects. An ideal agent for sedation during endoscopy should have rapid onset, ongoing effects only during the endoscopic procedure, quick recovery, and few side effects. However, today there is no ideal agent with all these features. The present study aimed to identify a near-ideal agent for UGIS endoscopy. Therein lies the originality of the present study.

It is known that propofol can induce arterial hypotension and respiratory depression due to sympathetic nervous system inhibition and direct vasodilator effects.^[12]^ Dexmedetomidine provides adequate analgesia, better hemodynamic stability, and does not cause respiratory depression at therapeutic doses. It is useful effects optimize dexmedetomidine for conscious sedation.^[13]^ In the present study, dexmedetomidine was combined with propofol to create ideal sedation levels and provide rapid recovery. Due to the sympatholytic effects of both agents, the combination was worrying, but sedation was achieved with a fairly stable hemodynamics and a suitable depth. In the combination of propofol with ketamine, side effects of ketamine, such as increased secretion, vomiting, and hallucinations, were reduced with propofol, whereas ketamine supports propofol because of its analgesic feature.^[14]^ In our study, it was believed that in the ketofol group, statistically significant cough was associated with secretion increase, and short recovery time was associated with the short duration of propofol. Recently, the number of studies comparing other sedative agents used in endoscopy with dexmedetomidine has increased.^[15,16]^ In accordance with our results, Nishizawa et al^[17]^ determined that dexmedetomidine was safe and effective for gastrointestinal endoscopy in their meta-analysis. Whereas mild to moderate sedation dexmedetomidine is preferred, we believe it would be appropriate to add propfol in procedures that require long, painful, and deep sedation. However, unlike our study, El Mourad and his colleagues^[18]^ used the same agents for the sedation procedure in awake intubation. They reported rapid onset of sedation, shorter intubation time, more stable hemodynamics, less need for additional propofol, and increased anesthetist satisfaction using ketofol. They also reported fewer side effects with ketofol. In general, publications relating to dexmedetomidine have emphasized the decrease in heart rate.^[19–21]^ Abbas et al^[22]^ studied using dexmedetomidine + propfol, ketamine + propofol, and propofol only. They evaluated and recorded heart rate and mean arterial pressure changes. There was a significant decrease in heart rate in the propofol only group and a significant increase in mean arterial pressure in the ketamine + propofol group. In the dexmedetomidine group, they reported more stable hemodynamics in accordance with our study.

It is difficult to apply and maintain adequate sedation. The level of consciousness is routinely evaluated by experienced anesthesiologists by observing physiological parameters and patient response. Establishing an objective indicator to monitor the level of patient sedation is crucial. The depth of anesthesia can be measured using ASA clinical evaluation suggestions, such as the Modified Observer's Assessment of Alertness/Sedation (MOAA/S) Scale or the Modified RSS. Monitoring methods, such as BIS, can also be used.^[23]^ For this purpose, we used RSS for clinical evaluation in our study. In our study, there was no difference between the groups in our clinical evaluation of anesthesia depth. Earlier access to target sedation levels intended using anesthesia depth monitors can provide more effective titration of sedative and analgesic drugs. This provides a decrease in the risk of adverse events caused by excessive sedation (insufficient oxygenation or ventilation or circulatory disturbance) and safe sedation.^[24]^ It plays an important role in applying sedation using BIS to reduce the incidence of awareness risk for operations such as endoscopy. Regardless of the clinical features of the patient and the sedative drug used, the BIS score varies between 0 and 100 (0 = coma, 40–60 = general anesthesia, 60–90 = sedation, 100 = awake). In a meta-analysis evaluating BIS follow-up during endoscopic procedures, it was found that BIS follow-up did not cause a significant difference in compilation time compared to standard monitoring and provided a significant reduction in propofol consumption.^[25]^ In our study, BIS monitoring was performed, and the target range was kept between 60 and 80. There was no statistical difference in terms of BIS values between the two groups in the presedation period. The high satisfaction in Group D may have been associated with rapid sedation due to propofol and the respiratory protection and analgesic effect of dexmedetomidine. BIS can be more useful when a longer sedation time is required, such as therapeutic endoscopic procedures. BIS has been found effective in reducing anesthetic consumption and supporting postoperative recovery from relatively deep anesthesia.^[9]^ The lack of any need for additional propofol doses in both groups in the study may be associated with the use of BIS.

In this study, no statistically significant difference was found between the groups, but verbal satisfaction in Group D was high in terms of endoscopist satisfaction, patient satisfaction, and patient tolerance. This finding was similar with the results of Yagan et al^[26]^ and El Mourad et al,^[18]^ and it differed from Sruthi et al.^[27]^ We believe that the different results of Sruthi et al may be related to differences in the doses used.

Hypoxemia is the most common adverse event encountered with propofol sedation during endoscopic procedures.^[28]^ Although there was no difference between the groups in terms of SpO2 values according to pulse oximeter, there was a slight decrease in saturation in both groups compared to the presedation period. Pulse oximetry effectively detects oxygen desaturation in patients undergoing sedation and analgesia, and both ASA and ASGE recommend the use of pulse oximetry, as used in our study, during all sedation endoscopic procedures.^[7]^

This study has a few limitations. First, the study covered only low-risk patients (ASA I and II). Therefore, future studies should include patients with high risk (ASA III to ASA IV). Secondly, recovery times of patients after sedation were studied, but the time to reach the level of satisfaction sedation was not studied. Finally, this study was a single-center study, and results cannot be generalized.

In conclusion, the present study suggested that dexmedetomidine–-propofol combination has similar or sometimes superior effects with ketamine-propfol combinations. We showed that it can be used safely in sedation procedures performed during UGIS endoscopy. It was observed that the dexmedetomidine-propfol combination was superior in terms of sedation depth, and ketamine-propofol combination was superior in terms of early recovery. The combination of dexmedetomidine-propofol has come to the fore due to its hemodynamic stability and minimal side effects. In terms of SpO2, neither combination proved superior. However, a partial decrease in saturation was detected in both groups compared to the presedation period. While ketamine propofol combination is mostly preferred for sedation, we have shown that dexmedetomidine-propofol combination is an effective and reliable option for sedation in endoscopic procedures. For better sedation levels, safe hemodynamics, protection of oxygenation, and fewer side effects, dexmedetomidine-propofol combination can be recommended for upper gastrointestinal endoscopy sedation. Propofol anesthesia with BIS protocol could be safe and useful for therapeutic endoscopy under deep sedation with spontaneous respiration. However, it is recommended that these findings should be supported with future studies that include a larger number of centers and wider patient participation.

## Author contributions

**Conceptualization:** Arzu Esen Tekeli.

**Data curation:** Arzu Esen Tekeli, Ali Kendal Oğuz, Yunus Emre Tunçdemir, Necat Almali.

**Formal analysis:** Arzu Esen Tekeli, Necat Almali.

**Investigation:** Ali Kendal Oğuz, Necat Almali.

**Methodology:** Arzu Esen Tekeli.

**Resources:** Arzu Esen Tekeli, Ali Kendal Oğuz.

**Software:** Yunus Emre Tunçdemir.

**Validation:** Ali Kendal Oğuz.

**Visualization:** Yunus Emre Tunçdemir.

**Writing – original draft:** Arzu Esen Tekeli.

**Writing – review & editing:** Arzu Esen Tekeli.
